# *Nature Communications* at ten

**DOI:** 10.1038/s41467-020-15592-3

**Published:** 2020-04-14

## Abstract

*Nature Communications* launched in April 2010 with the mission to publish significant advances in each field in a multidisciplinary venue. Ten years on, we reflect on our achievements and look at future challenges in a changing publishing landscape.

For the past ten years, *Nature Communications* has been a pioneer within the Nature family in many ways. Launched as the 17th Nature-branded research title, it was the first to publish content online-only. It was also the first hybrid Nature-branded journal, offering an Open Access option to authors, before flipping to fully Open Access in 2016 [https://www.nature.com/articles/ncomms6523].

The journal began [https://www.nature.com/articles/464958a] life with the purpose to publish “rigorous and comprehensive papers that represent advances of significance to specialists within each field”, as well as to serve communities that don’t have a dedicated home in the Nature family or researchers seeking publication in a broad journal. Another founding principle was to offer a platform for multidisciplinary works [https://www.nature.com/articles/ncomms1011], given that, “the number of truly multidisciplinary primary research journals can be counted on one hand”. We wish to continue to meet those needs for authors and readers.

“*Nature Communications* has taken pride in being an early adopter of initiatives aimed at increasing transparency and promoting open science”

*Nature Communications* has taken pride in being an early adopter of initiatives aimed at increasing transparency and promoting open science. In 2016 we offered our authors the chance to publish the peer review history [https://www.nature.com/articles/ncomms10277]—reviewer reports and author responses—alongside their paper. The uptake [https://www.nature.com/articles/ncomms13626] was encouragingly high at over 60% in the first year, and it increased to 69% in 2019. A number of other Nature-branded journals [https://www.nature.com/articles/d41586-020-00309-9] have recently followed suit, highlighting the value that transparent peer review brings to our readership.

Then, in 2017, we started an initiative [https://www.nature.com/articles/s41467-017-00950-5] aimed at giving greater visibility to research results during the peer review process by offering authors the option to link the preprint of papers under review at our journal on a dedicated website. Nature Research has long supported preprint servers, and now actively encourages [https://www.nature.com/articles/d41586-019-01493-z] their use. We believe that our commitments show how journals and preprint servers can better work together to widely and rapidly disseminate scientific results.

Transparent and open practices ultimately aim at making research results more reproducible and trustworthy, and Nature-branded journals strive to contribute in raising standards. Data sharing plays an important role in reproducibility. For specific datasets, we mandate deposition in a public repository at acceptance and availability to reviewers. Since 2018 we strongly encourage that all raw data associated with a paper be made available at acceptance [https://www.nature.com/articles/s41467-018-05227-z] and during review [https://www.nature.com/articles/s41467-018-06012-8]. We now request that relevant reproducibility checklists are completed by authors at the start of the peer review process.

The great work done by our authors is strengthened by the input received from peer reviewers, who are often the unsung heroes of peer review. Starting in 2018, we joined *Nature* and other sister journals in offering our reviewers the option of being named on the papers they review to get recognition [https://www.nature.com/articles/d41586-019-01162-1]. So far, 32% of reviewers have agreed to be named, while 19% have signed their reports.

Ten years on and 27,833 published Articles later (as of 1st of April), *Nature Communications* is the leading Open Access journal from Springer Nature. Over the years, we built the world’s largest team of professional editors for a scientific journal, currently 101 strong and spread over four countries and three continents. Our team is highly diverse, holding 23 nationalities; 56% of our editors are women. Our editors’ range of expertise across the physical, life, Earth and social sciences ensures that we are uniquely equipped to handle papers across a vast variety of topics. Furthermore, the close cooperation between editors with different expertise allows for the proper assessment of multidisciplinary work from all relevant scientific aspects.Our editors are our strength: they are embedded in their research communities which they champion in our journal.

Diversity is essential in ensuring the robustness of scientific endeavour. In 2019, 17% of our reviewers were female and 69% male (the rest didn’t provide gender information). We are committed to achieving a greater gender balance in 2020 and beyond, both in our reviewer pool and in our commissioned content. We also want to be more inclusive of junior researchers in peer review and we are planning initiatives to foster the training of the next generation.

Looking to the future, we will continue to expand the breadth of content that we cover. We are increasingly interested in applied work, especially those areas that address issues of societal challenge, such as climate, energy, food and health. We are also expanding our outreach in the clinical and social sciences. We wish to offer a platform for influential studies in all areas of medical research, from translational medicine to clinical practice. We are particularly interested in publishing clinical trials, including trials with negative results as we recognise the importance of such reports for the progress of medicine, as well as pilot and feasibility trials.

We will also continue to promote reproducibility, and we will start offering our authors the Registered Reports format. This article type allows authors to have their study design protocol peer-reviewed before they begin their investigations. Once this initial review is done, we will commit to publishing the report once the study is completed, regardless of the results obtained. This format underlines the importance of the research question and the quality of the study plan as considerations for publication. We hope that this article type will stimulate more researchers to embrace replication studies for questions of high importance.

We have set up a dedicated anniversary site [https://promo.nature.com/ncomms-10year/] to showcase content that celebrates our identity and the exciting science we publish, to be updated throughout 2020. In a series of paired Comments, we will highlight what the past ten years have meant for a number of research fields, and what is on their horizon for the next ten. Authors of our very first papers will share their thoughts and experiences of publishing with *Nature Communications* as a new, online-only journal; we sought insights from our longest serving editor on the journal evolution over the past ten years; finally, our current and past Chief Editors and Editors-in-Chief will discuss what makes a journal like *Nature Communications* unique and why multidisciplinary journals are so important in our complex world.

As mentioned above, we are keen to support junior researchers. We will be launching a competition on Twitter for early career researchers to share their experience of publishing in our journal.The top entries will be published on our anniversary website. Last but not least, we will tell the story of the people who are *Nature Communications:* our editors, our support staff and production team. Watch this space!

We wish to warmly thank our past and present supporters—authors, reviewers, readers and editors—without which *Nature Communications* would not be as successful as it is. We are proud of what we have achieved together over the past ten years and look forward to what we will achieve in the next ten.

